# Correlation between chest CT severity scoring system with oxygen saturation and laboratory inflammatory markers in adult patients with COVID-19 infection

**DOI:** 10.1186/s43055-022-00747-7

**Published:** 2022-03-17

**Authors:** Ahmed Gamil Ibrahim Abd El Megid, Mohamed El Shabrawy, Ahmed Abd El-Hamid Mohamed Abdalla

**Affiliations:** 1 Radiology Department, Faculty of Medicine, Zagazig University, Zagazig City, Sharkia Governorate Egypt; 2 Chest Department, Faculty of Medicine, Zagazig University, Zagazig City, Sharkia Governorate Egypt

**Keywords:** Computed tomography, COVID-19, CT severity score, Oxygen saturation

## Abstract

**Background:**

COVID-19 pneumonia is responsible for the latest pandemics. Chest computed tomography (CT) scan is known to be an essential tool for diagnosis of COVID-19. In this research, the relationship between on-admission chest CT severity score, capillary blood oxygen saturation level, and laboratory inflammatory markers results in patients with SARS-COV-2 pneumonia was investigated.

**Methods:**

This prospective analytical study was conducted in COVID-19 isolation unit, Zagazig University Hospitals, from 1st to end of April 2021. Adult patients with COVID-19 infection were included. Chest CT scan was performed for all patients, and CT severity score was computed. The initial capillary oxygen saturation was also assessed at the time of admission. The information was gathered and analyzed.

**Results:**

A total number of 305 COVID-19 patients were involved in the study with the following data: age, gender, presence of co morbidities, capillary blood oxygen saturation, laboratory tests including absolute lymphocytic count, CRP, D-dimer and ferritin levels, as well as chest CT severity score. Based on chest CT severity score, we found that 110 cases (36.1%) were mild, 163 cases (53.4%) were moderate, and 32 cases (10.5%) were severe, with significant male predominance among moderate and severe cases. The initial measurements of blood oxygen saturation values revealed that mean blood oxygen saturation was 95.6% among mild to moderate cases and 85.4% among severe cases. Furthermore, there was a high statistically significant negative correlation between chest CT severity score and absolute lymphocytic count of studied cases, while there was a statistically significant positive correlation with D-dimer, CRP and ferritin levels.

**Conclusions:**

CT scans can help clinicians in developing a management strategy and serve as a predictor of illness severity and possible outcomes. In individuals with COVID-19 infection, the severity of a chest CT scan is positively correlated to inflammatory markers and oxygen demand.

## Introduction

The world's attention was drawn to a novel human coronavirus, SARS-CoV-2 (severe acute respiratory syndrome coronavirus-2), after an outbreak of unexplained pneumonia in Wuhan, China, in December 2019, the potentially fatal disease caused by SARS-CoV-2, was declared a pandemic by the World Health Organization in March 2020 [1].

COVID-19 is an emerging disease. It induces interlobular septal edema as well as lymphocyte infiltration in the pulmonary interstitium. Despite the fact that early airspace and alveolar exudate accumulation are minor, the condition can rapidly worsen [2].

Fever, tiredness, dry cough, myalgia, shortness of breath, and gastrointestinal problems as diarrhea and vomiting are the most prevalent clinical presentations of COVID-19 pneumonia [3, 4]. According to the evidence, the presentations of the disease vary among individuals, but the main presentations include cough, upper airway inflammation, myalgia, headache, acute respiratory distress syndrome (ARDS), decreased blood O_2_ saturation, and lungs involvement in imaging investigations [5]. These lung changes have also been reported to be the most critical prognostic variables in COVID-19 infected patients [5].

The reverse transcription-polymerase chain reaction (RT-PCR), which has a high specificity, is the gold-standard approach for COVID-19 diagnosis. Still, the downside of this tool is its lack of sensitivity and the lengthy time it takes to establish a diagnosis [6]. Chest computed tomography (CT) is, on the other hand, a simple and quick method [7]. Despite having a very high sensitivity, which can reach up to 97.2% [8], CT has a very low specificity, which can be as low as 25% [7]. Laboratory data are also crucial in the diagnosis and management of COVID-19 patients [9].

The chest computed tomography (CT) scan is a useful tool in the identification of pulmonary changes, as well as in the screening, diagnosis, and clinical classification of patients suspected of infections [10].

Physicians can also use CT scans to follow up patients after they have been discharged from the hospital. CT scans have been utilized to describe the imaging findings of COVID-19-induced lung infection. Bilateral and multilobar ground-glass opacities are the most common infection patterns [11, 12]. The peripheral and lower lobes of the lung have also been proven to be the mostly affected regions. For patients infected with COVID-19, other radiological abnormalities such as crazy paving pattern, airway alteration, and reverse halo sign were discovered [13, 14].

Some researches have also highlighted the link between chest CT scan observations and a patient's clinical status, implying that a chest CT scan may play a role in identifying the degree and extent of the disease. The CT severity score system is a semi-quantitative scoring system designed to assess the severity and scope of the viral pneumonia lung involvement [15]. The scoring system could be utilized to estimate the extent of COVID-19 pneumonia in patients' lungs as well as to assess their clinical status [16].

Almost all patients with COVID-19 infection admitted to the hospitals, blood oxygen saturation measured with a pulse oximetry instrument [17]. Hypoxia may be detected in patients with this instrument indicating that the condition is more severe and that intensive care is required [18].

## Methods

**Fig. 1:**
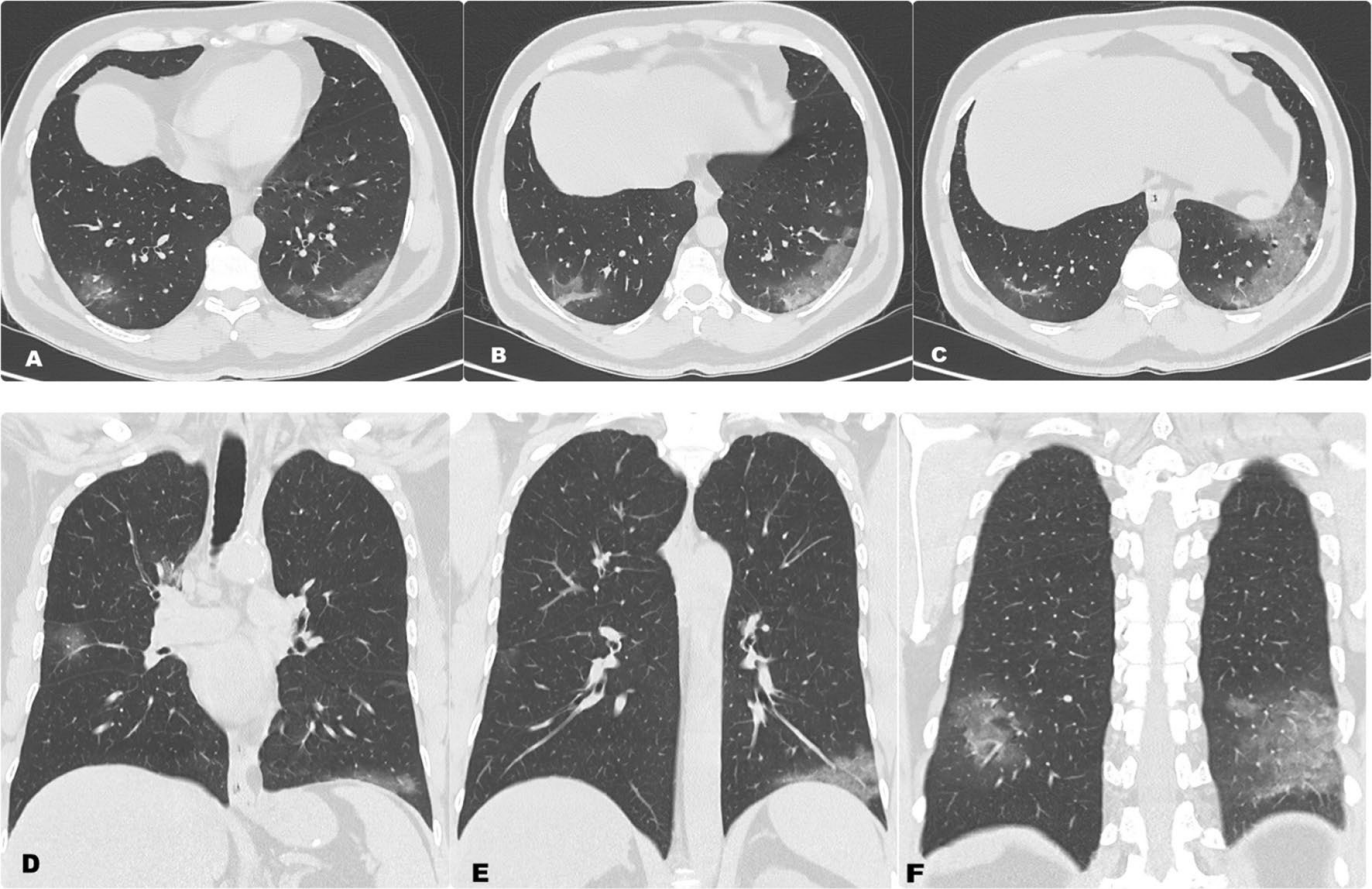
**A**–**C** Axial and **D**–**F** reformatted coronal non-contrast CT chest of a 56-year-old male patient with positive RT-PCR for Covid-19, show bilateral scattered ground-glass opacities with a primarily peripheral distribution. CT severity score is 7 points which corresponds to mild involvement

**Fig. 2:**
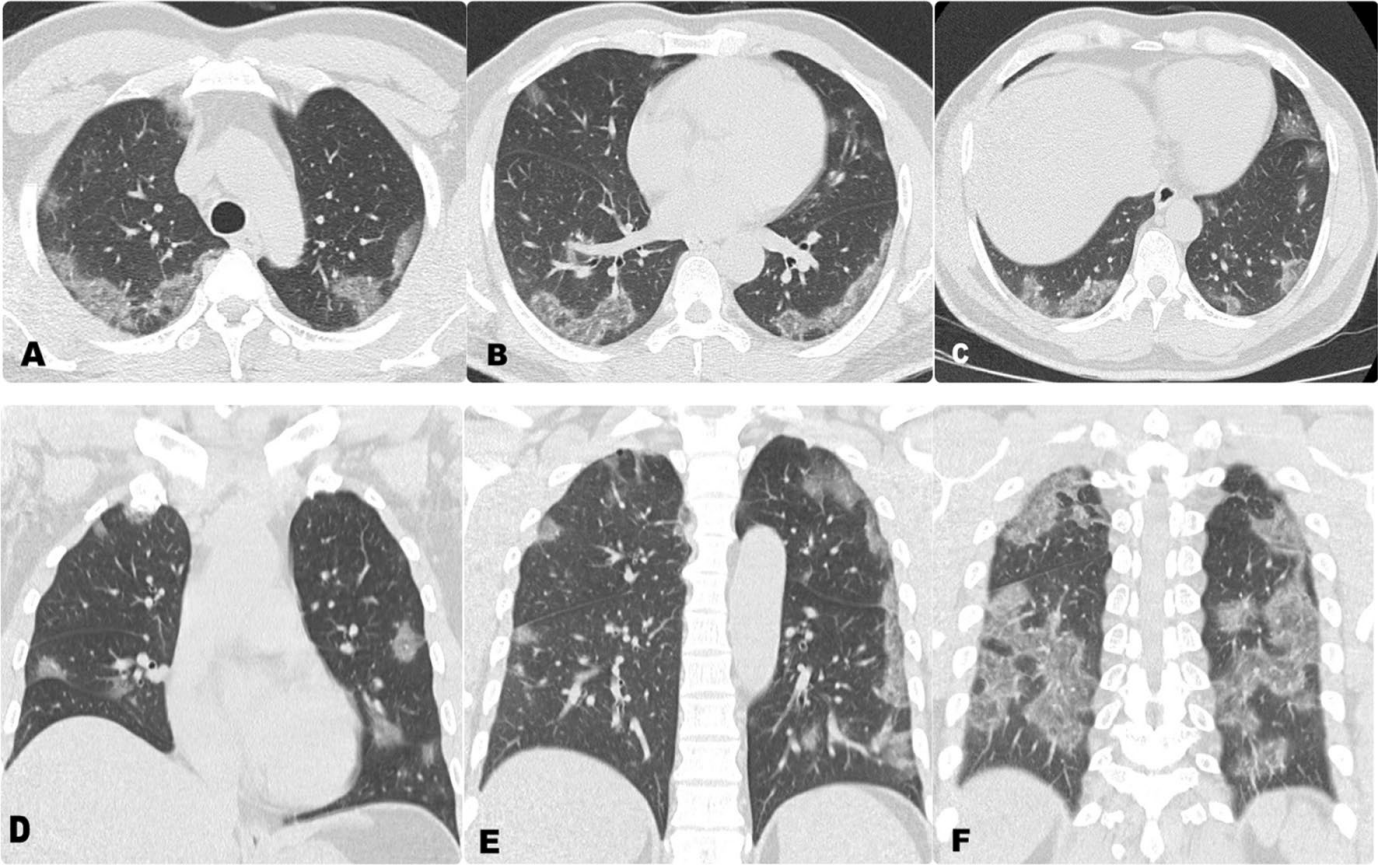
**A**–**C** Axial and **D**–**F** reformatted coronal non-contrast CT chest of a 53-year-old male patient with positive RT-PCR for Covid-19, show bilateral scattered ground-glass opacities with a primarily peripheral distribution. CT severity score is 15 points which corresponds to moderate involvement

This prospective analytical study was conducted in COVID-19 isolation unit, Zagazig University Hospitals, Egypt in the period from 1st to end of April 2021. The approval of this prospective study was obtained from the institutional review board and written consents from all subjects were achieved (Figs. 1 and 2).

**Fig. 3:**
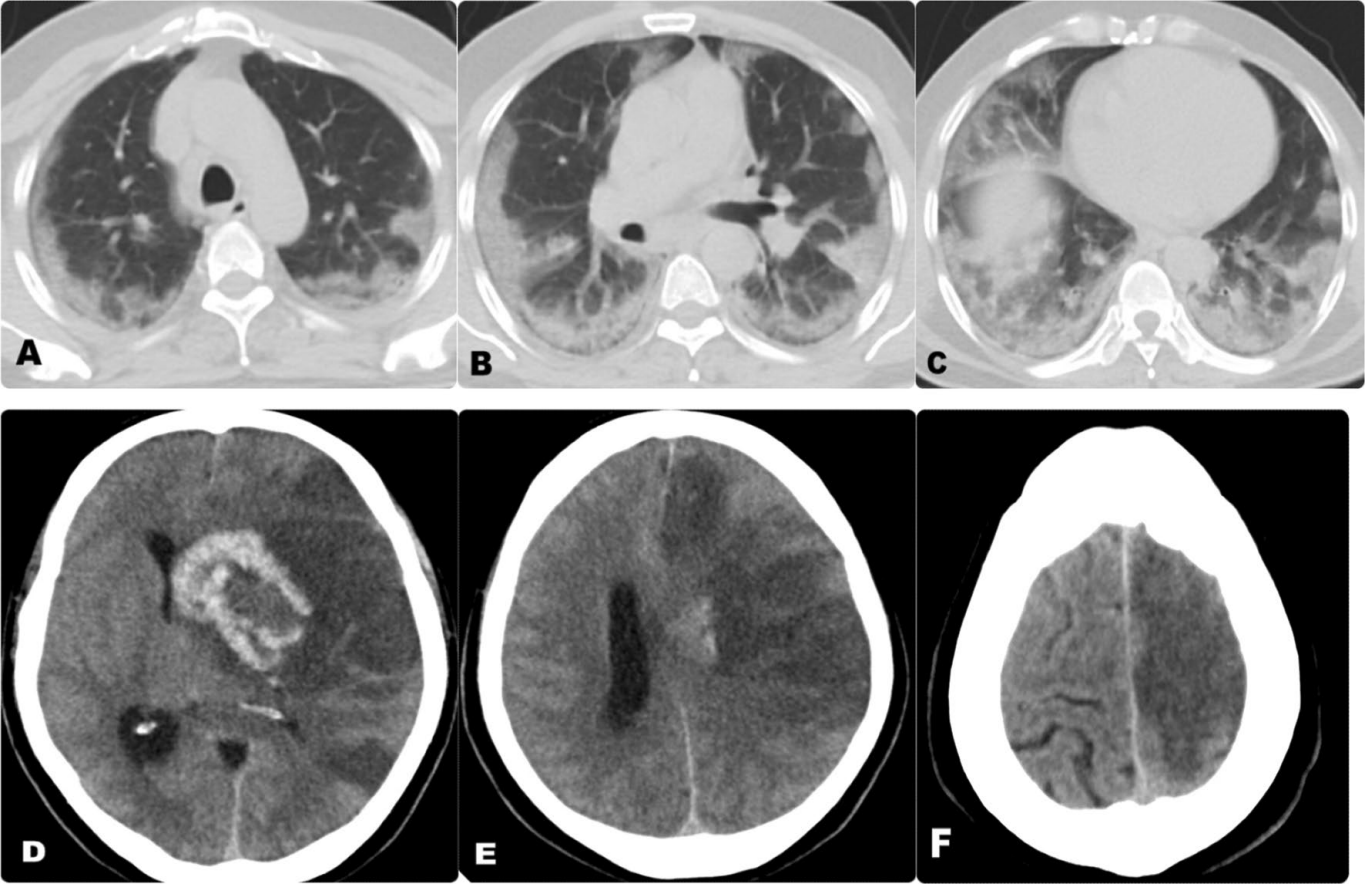
**A**–**C** Axial non-contrast CT chest of a 42-year-old male patient with positive RT-PCR for Covid-19, show bilateral scattered ground-glass and air-space consolidation opacities with a primarily peripheral distribution. CT severity score is 19 points which corresponds to sever involvement. **D**–**F** Axial non contrast CT brain for the same patient, showing hemorrhagic infarction at the left fronto-parietal region, exerting mass effect in the form of compression of the related lateral ventricle and mild mid line shift

**Fig. 4:**
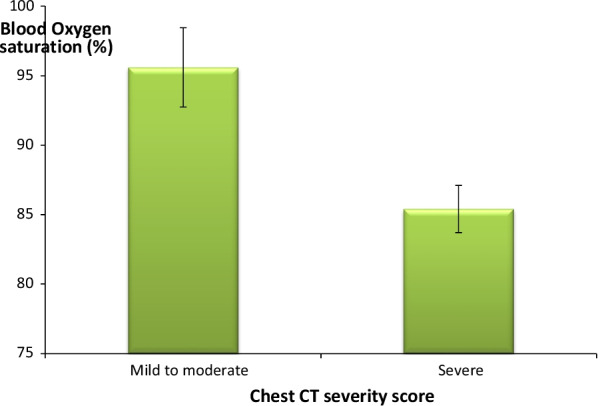
Difference in blood oxygen saturation in relation to grading of chest CT severity score

The inclusion criteria were adult patients (18 years old or more), confirmed to have COVID-19 infection by PCR testing, patients who underwent chest CT scan, and measurements of capillary oxygen saturation using pulse oximetry at the time of admission, and having written informed consent to participate in the research (Figs. 3 and 4).

**Fig. 5:**
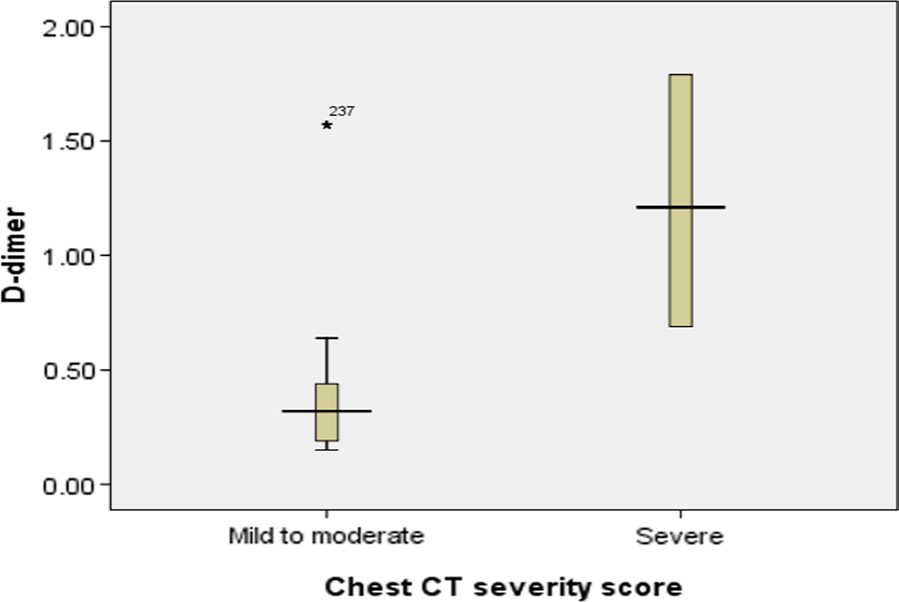
Box-plot analysis of D-dimer in relation to grading of CT severity score

**Fig. 6:**
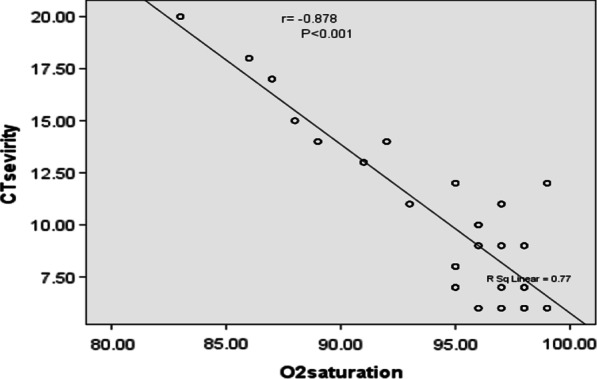
Negative correlation between chest CT severity score and oxygen saturation level among studied cases

**Fig. 7:**
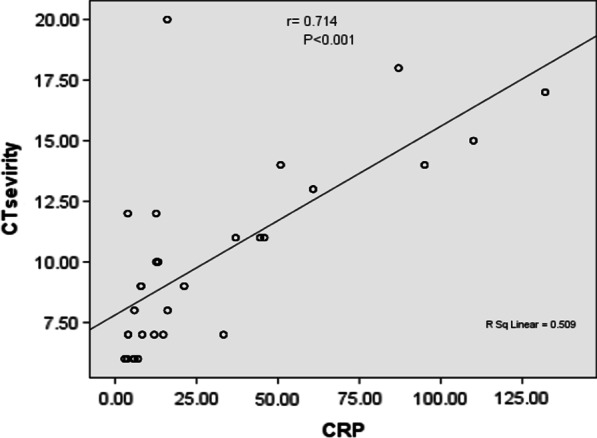
Positive correlation between CT severity index and CRP level among studied cases

The exclusion criteria were patients less than 18 years old, patients not confirmed to have COVID-19 infection by PCR testing, patients who did not undergo chest CT scan, patients with unavailable clinical and laboratory data, congenital lung and heart anomalies, and patients with severe hemoglobinopathy or anemia (Figs. 5, 6 and 7).

In this study, the relationship between on-admission chest CT severity score, capillary blood oxygen saturation level, and laboratory inflammatory markers results in adult positive PCR patients with SARS-COV-2 pneumonia was investigated.

All subjects underwent chest CT scan by 128 multi detectors device (Ingenuity Phillips health care), scanning parameters were as follows: scan orientation (craniocaudally), tube voltage (120 kV), tube current (100–600 mA)-smart mA dose modulation, slice collimation (128 × 0.625 mm), width (0.625 × 0.625 mm) and were in supine and deep inspiration. After that, the CT scan images were reassembled.

Two competent radiologists assessed all patients' chest CT scans at the same time and calculated the chest CT severity score for patients (a competent radiologist is a radiologist who has more than 5 years of experience in the field of thoracic imaging). In cases of disagreement, they enlisted the expertise of a third radiologist with more experience in thoracic imaging (more than 8 years).

All pulmonary lobe segments were investigated for the existence and spread of parenchymal abnormalities, like ground-glass opacities, consolidation, crazy paving and pulmonary nodules. Chest CT severity score was assigned for each lobe as follow: 0 for no involvement, 1 for < 5% involvement, 2 for 5–25% involvement, 3 for 25–50% involvement, 4 for 50–75% involvement, and 5 for > 75% involvement. The overall severity score got by summation of scores from all five lung lobes. A mild grade is of 0–7 points, a moderate grade is of 8–16 points, and a severe grade is of 17–25 points.

The sum of points in each lobe was used to calculate the final score, which was used to calculate the CT severity score.

The capillary blood oxygen saturation of all patients was measured and acquired using pulse oximetry. It should also be mentioned that the time interval between performing pulse oximetry and chest CT scan was no more than 1 day because of possible lung and blood oxygen saturation changes with time.

Other clinical information and laboratory results like absolute lymphocyte count, CRP, D-dimer and ferritin levels were also gathered from documents of all patients.

Eventually, 305 patients were included with the following data been collected: age, gender, presence of comorbidities/risk factors, blood oxygen saturation, laboratory results including absolute lymphocyte count, CRP, D-dimer and ferritin levels.

After data collection, it was analyzed to investigate the relationship between chest CT severity score with patient’s capillary blood oxygen saturation values and laboratory inflammatory markers results using IBM SPSS 23.0 for windows. Quantitative data were expressed as mean ± standard deviation (SD). Qualitative data were presented as frequency and percentage.

The following tests were done:

- T-test of significance was used when comparing between two means of normally distributed data.
- Mann Whitney U test was used when comparing two means of not normally distributed data.
- Chi-square (*X*^2^) test of significance was used in order to compare proportions between two qualitative parameters.
- Pearson`s correlation test was used for comparing two continuous quantitative data. Probability (*P* value): *P* value ≤ 0.05 was considered significant, *P* value ≤ 0.001 was considered as highly significant and *P* value > 0.05 was considered insignificant.

## Results

Three hundred and five (305) patients with confirmed covid infection were enrolled in this study; where their mean age was 41.9 ± 9.1 years [range 18–73 years; 202 males (66.2%), and 103 females (33.8%)].

The risk factors considered included diabetes mellitus, hypertension, chronic cardiac disease, chronic kidney disease and asthma. Risk factors were found in 124/305 patients (40.7%), 90 patients had more than one risk factor (Table 1).

**Table 1:** General characteristics of the studied cases

Variables	Cases (*N* = 305)
	Mean ± SD
Age	41.9 ± 9.1 (18–73)
Sex	*N* (%)
Male	202 (66.2%)
Female	103 (33.8%)
Co-morbidities	
No	181 (59.3%)
Diabetes mellitus (DM)	76 (24.9%)
Hypertension	86 (28.2%)
Chronic cardiac disease	25 (8.2%)
Chronic kidney disease	9 (3.0%)
Asthma	9 (3.0%)
> 1 disease present	90 (29.5%)

The clinical presentation varied. The most common clinical symptoms were fever (162/53.1%) and cough (121/39.7%) and the least was cerebral stroke in one patient. Note that some patients presented with more than one clinical parameter (Table 2).

**Table 2:** Clinical presentation of the studied cases

Variables	Cases (*N* = 305)
	*N* (%)
Cough	121 (39.7%)
Difficulty in breathing	27 (8.9%)
Fever	162 (53.1%)
Sore throat	63 (20.7%)
Body aches	112 (36.7%)
Chest pain	25 (8.2%)
GIT symptoms	37 (12.2%)
Cerebral stroke	1 (0.3%)

### Chest CT severity score and clinical parameters correlation:

In our study, we found that 110 cases (36.1%) were mild, 60 from them were male and 50 were female, 163 cases (53.4%) were moderate, 121 from them were male and 42 were female and 32 cases (10.5%) were severe, 21 from them were male and 11 were female. Male gender was statistically significant correlated with moderate to severe chest CT severity scores (Tables 3 and 4) (Figs. 1, 2 and 3).

**Table 3:** CT severity score among the studied cases

Variables	Cases	Gender	
	(*N* = 305)	Male	Female
	*N* (%)		
Mild	110 (36.1%)	60	50
Moderate	163 (53.4%)	121	42
Severe	32 (10.5%)	21	11

**Table 4:** Relation between chest CT severity score and gender

	Chest CT severity score			Total	*P*
	Mild	Moderate	Severe		
*Sex*				11.39	0.003 S
Male					
*N*	60	121	21		
% Within sex	29.7%	59.9%	10.4%		
% Within score	54.5%	74.2%	65.6%		
Female					
*N*	50	42	11		
% Within sex	48.5%	40.8%	10.7%		
% Within score	45.5%	25.8%	34.4%		

This table shows that male gender was statistically significant correlated with moderate and severe chest CT severity scores

The initial blood oxygen saturation measurements revealed that the mean blood oxygen saturation was 95.6% among mild to moderate cases and 85.4% among severe cases according to CT severity score (Normal blood oxygen saturation level is 95% or higher).

There was a high statistically significant negative correlation between CT severity score and blood oxygen saturation level (Fig. 4) (Table 5).

### Laboratory results:

Mean absolute lymphocytic count was 1.53 × 10^9^/L among mild to moderate cases and 1.18 × 10^9^/L among sever cases according to CT chest severity score (normal value 1.5–4 × 109/L). Mean D-dimer was 0.38 mg/L among mild to moderate cases and 1.23 mg/L among sever cases (Normal value is less than 0.50). Mean CRP was 24.7 mg/L among mild to moderate cases and 80.3 mg/L among sever cases (Normal value is less than 10 mg/L). Mean Ferritin level was 551 micog/L among mild to moderate cases and 926.8 microg/L among sever cases (Normal range 12–300 microg/L for male and 12–150 microg/L for female) (Table 4).

There was a high statistically significant negative correlation between CT severity score and absolute lymphocytic count of studied cases, while there was a statistically significant positive correlation with D-dimer, CRP and ferritin levels (Table 5) (Figs. 5, 6 and 7).

**Table 5:** Relation between chest CT severity score and laboratory data of the studied cases

	Grouping of chest CT severity score	*N*	Median range	Mean	S.D	Test	*P*
Blood oxygen saturation level	Mild to moderate	273	96 88–99	95.6	2.85	19.8*	< 0.001 HS
	Severe	32	86 83–87	85.4	1.7		
Absolute lymphocytic count	Mild to moderate	273	1.5 0.51–2.9	1.53	0.56	4.11^#^	< 0.001 HS
	Severe	32	1.12 1.1–1.3	1.18	0.09		
D-dimer	Mild to moderate	273	0.32 0.15–1.57	0.38	0.29	8.79^#^	< 0.001 HS
	Severe	32	1.21 0.69–1.79	1.23	0.46		
CRP	Mild to moderate	273	12.6 3–110	24.7	28.3	6.57^#^	< 0.001 HS
	Severe	32	87 16–132	80.3	47.9		
Ferritin	Mild to moderate	273	490 98–1463	551.01	341.5	4.69^#^	< 0.001 HS
	Severe	32	687 558–1502	926.8	426.2		

This table shows that blood oxygen saturation level and absolute lymphocytic count was statistically significantly higher among cases with mild to moderate CT severity index, while D-dimer, CRP and ferritin level was statistically significantly higher among cases with severe chest CT severity score

HS: *P* value < 0.001 is high significant

*t-test of significance

^#^Mann–whitney test for non-parametric data

## Discussion

In our study, we illustrated the role of chest CT and chest CT severity score in evaluation of the adult patients with COVID-19, proved by PCR nasal swab, and their relations with initial blood oxygen saturation level and other laboratory inflammatory markers results (Table 6).

**Table 6:** Correlation between chest CT severity score and laboratory data of the studied cases

Laboratory data	Test	CT severity score *N* = 305
Oxygen saturation	R	− 0.878**
	P	< 0.001 HS
Absolute lymphocytic count	R	− 0.263
	P	< 0.001 HS
D-dimer	R	0.633**
	P	< 0.001 HS
CRP	R	0.714**
	P	< 0.001 HS
Ferritin	R	0.498**
	P	< 0.001 HS

** means high significant

This table reveals that CT severity score, blood oxygen saturation level, and absolute lymphocytic count of examined cases had a high statistically significant negative correlation, but D-dimer, CRP, and ferritin had a statistically significant positive correlation

Our population revealed a relatively young age (mean 41.9 years) with male inclination (66.2%). Severe disease was mostly seen in males (65.6%).

Studies suggest that such distribution can be attributed to a variety of variables, including behavioral differences and the potential protective impact of estrogen [19]**.**

The risk factors considered in our study, included diabetes mellitus, hypertension, chronic cardiac disease, chronic kidney disease and asthma. Risk factors were found in 124/305 patients (40.7%), 90 patients had more than one risk factor.

According to Guan et al., the existence of risk factors, such as hypertension, diabetes, lung illness, and coronary artery disease, is associated with a bad prognosis, with an even poorer outcome when several risk factors are present [20].

On chest CT, each of the five lung lobes was assessed visually. The severity levels were then divided into categories depending on the total cumulative severity score.

Our findings revealed that there was a significant reverse relationship between chest CT severity score and blood oxygen saturation level, which is crucial in clinical practice. These findings emphasize the relationship between chest CT severity score and clinical status of patients with COVID-19 infections.

Quantitative and semi-quantitative markers of chest CT scan and their association to patient clinical circumstances were investigated in a study by Yang et al. [16] in China. They looked at the CT scan results of 102 individuals with COVID-19 infection and discovered that patients with severe COVID-19 infections had a significantly higher total CT severity score than those with moderate infections. A CT severity score could potentially be used to assess the severity of lung involvement, according to the research. Resting blood oxygen saturation of less than 93% was also considered a clinical severity criterion. These data support our findings, showing higher CT severity scores in patients with hypoxia.

Lymphopenia was found to be associated with an increasing chest CT severity score. Tavakolpour et al. [21], stated that presence of lymphopenia can be related to the inflammatory cytokine storm. In extreme cases, T cell counts, particularly CD8+, have been found to be lower.

Furthermore, our findings showed that the serum CRP level had a strong association with chest CT severity score. Similarly, serum ferritin is an important mediator of immune dysregulation, and its level was closely linked to illness severity. D-dimer likewise can be used as a prognostic indicator, where higher levels are seen in more critical conditions.

Hypoxic patients have significantly higher CT severity scores, according to our findings. We conclude that there was a significant reverse relationship between CT severity score and blood oxygen saturation level which has major clinical implications. We suggest that in patients with COVID-19 infection, clinicians should pay closer attention to chest CT severity scores.

### Limitations

This study has limitations. First, small sample size, so a bigger multicenter cohort with a larger sample size is required to improve the accuracy of the results. Second, the reality that the assessment of disease severity on CT scans can be subjective. This was mitigated by incorporating two or more experienced readers to reach a consensus. Finally, the other factors that might contribute to the disease severity as lifestyle and comorbidities/risk factors should be considered in more details, so further investigations and researches are needed.

## Conclusions

Chest CT scans can help clinicians in developing a management strategy and serve as a predictor of illness severity and possible outcomes. In individuals with COVID-19 infection, the severity of a chest CT scan is positively correlated to inflammatory markers and oxygen demand.

## Data Availability

The datasets used and/or analyzed during the current study are available from the corresponding author on reasonable request.

## Abbreviations

ARDS: Acute respiratory distress syndrome
COVID-19: Corona virus disease of 2019
CRP: C reactive protein
CT: Computed tomography
DM: Diabetes mellitus
O_2_: Oxygen
RT-PCR: Reverse transcription-polymerase chain reaction
SARS-CoV-2: Severe acute respiratory syndrome corona virus-2
